# Phytochemical constituents, antioxidant activity, and antiproliferative properties of black, red, and brown rice bran

**DOI:** 10.1186/s13065-018-0382-9

**Published:** 2018-02-17

**Authors:** Ali Ghasemzadeh, Mohamad Taghi Karbalaii, Hawa Z. E. Jaafar, Asmah Rahmat

**Affiliations:** 1 Department of Crop Science, Faculty of Agriculture, Universiti Putra Malaysia, 43400 Serdang, Selangor Malaysia; 2 Department of Land Management, Faculty of Agriculture, Universiti Putra Malaysia, 43400 Serdang, Selangor Malaysia; 3 Agricultural Research Extraction and Education Organization, Rice Research Institute of Iran, Amol, Mazandaran Iran; 4 Department of Nutrition & Dietetics, Faculty of Medicine & Health Sciences, Universiti Putra Malaysia, 43400 Serdang, Selangor Malaysia

**Keywords:** Rice bran, Phytochemicals, Antioxidant activity, Antiproliferative activity, Black rice bran, Flavonoids

## Abstract

**Background:**

In the recent years, the health benefits of the pigmented rice varieties have reported due to the presence of bioactive compounds. In this study, the phytochemical constituents (total phenolic, flavonoid and anthocyanin content) and individual phenolics and flavonoids of the extracts of sixteen genotypes of pigmented rice bran were evaluated using spectrophotometric and ultra-high performance liquid chromatography method. Antioxidative properties of the free and bound fractions were evaluated using nitric oxide and 1,1-diphenyl-2-picrylhydrazyl scavenging assays. Extracts were evaluated for antiproliferative activity against breast cancer cell lines (MCF-7 and MDA-MB-231) using the MTT assay.

**Results:**

Signifficant diferences were observed in the concentrations of phytochemicals and biological activities among different pigmented rice brans. The highest phytochemical content was observed in black rice bran followed by red and brown rice bran. The concentration of free individual flavonoids and phenolic compounds were significantly higher than those of bound compounds except those of ferulic acid and *p*-coumaric acid. Highest antioxidant activities were observed in black rice bran, followed by red and brown rice bran extracts. Extracts of black rice bran exhibited potent antiproliferative activity, with half maximal inhibitory concentrations (IC_50_) of 148.6 and 119.2 mg/mL against MCF-7 and MDA-MB-231 cell lines, respectively, compared to the activity of the extracts of red rice bran (175.0 and 151.0 mg/mL, respectively) and brown rice bran (382.3 and 346.1 mg/mL, respectively).

**Conclusions:**

Black rice bran contains high levels of phytochemicals, and thus has potent pharmaceutical activity. This highlights opportunities for researcher to breed new genotypes of rice with higher nutritional values, which the food industry can use to develop new products that will compete in expanding functional food markets.

## Background

Rice (*Oryza sativa* L.) is the staple food in several countries especially in Asian. Rice grains have a hard husk protecting the kernel inside. After the husk is removed, the remaining product is known as brown rice. After removal of the bran and embryo, the remaining endosperm is known as polished rice. Traditionally, polished rice is consumed. However, the rice bran fraction contains high levels of fibre and bioactive phytochemicals including tocopherols, tocotrienols, oryzanols, dietary fibres, vitamins, and phenolic compounds, which are beneficial to human health and well-being [1]. These phytochemicals are distributed in free, soluble-conjugated, and bound forms in the endosperm and bran/embryo fractions of the whole rice grain. Some studies have focused on whole and brown rice [2, 3] while others have investigated the bran fractions [4, 5] or endosperm fractions alone [6]. Another study has reported data on the husk, bran, and endosperm of rice [7].

According to the World Health Organization (WHO), breast cancer is the second-leading cause of death in women with 522,000 related deaths estimated in 2012 [8]. Therefore, breast cancer prevention and related therapeutic modalities are challenging areas of research. Phytochemicals are naturally occurring compounds found in crops and herbs, which provide health benefits for humans beyond those attributed to macronutrients and micronutrients [9]. The most important groups of phytochemicals found in whole grains can be classified as phenolics, carotenoids, vitamin E compounds, lignans, β-glucan, and inulin [10]. Phenolics are the products of secondary metabolism in plants and exert beneficial effects on human health [11]. Phenolics, one of the most abundant groups of phytochemicals in whole grains, are considered natural antioxidants, which act as radical scavengers to decrease the incidence of oxidative stress-induced damage to large biological molecules, such as lipids, proteins, and DNA [12]. An extraction procedure mainly for free phenolics was used on the milled fractions of rice for determining their antioxidant activities [13]. Such an extraction procedure may lead to underestimating the total phenolics and antioxidant activity if the bound fraction has not been included [7]. Thus, it can be seen that a direct comparison of the distribution of free and bound phytochemicals (phenolics and flavonoids) in different milled fractions (bran/embryo and endosperm) is complicated. Most of previous studies focused on phenolic acid content of rice and there is little information regarding flavonoids compounds and their concentration in free and bound fraction of different colour rice varieties. Pengkumsri et al. [14] and Moko et al. [15] compared phytochemical constituent and antioxidant activity of black, red and brown rice bran. They found that black rice bran with highest content of phytochemicals represent valuable antioxidant activity. Anti-tumor [16] and anti-inflammation activity [17] of black rice bran was reported by previous studies. One of the main obstacles is all of these studies evaluated free fraction of phytochemicals in pigmented rice, whereas, the moieties of phenolics (e.g. ferulic acid) and flavonoids (e.g. kaempferol, quercetin) in plants/crops are mainly in bound form. However, phytochemical synthesis of rice genotypes can be adversely affected under certain conditions or different varieties and following that pharmaceutical value will change. So far, however, there has been little discussion about characterization of the pigmented rice genotypes in terms of composition of free and bound secondary metabolites, as well as pharmaceutical aspects. This study provides new insights into free and bound composition of secondary metabolites in pigmented rice genotypes associated with antioxidant and antiproliferative activities.

The objectives of this study were: (1) to investigate the distribution of free and bound phenolics and flavonoids compounds in bran fractions of brown, red and black rice; and (2) to separate and identify of individual flavonoids and phenolic acids; and (3) to determine antioxidant and antiproliferative activity in bran fractions of brown, red and black rice.

## Methods

### Rice samples

Sixteen pigmented rice genotypes were grown in glasshouse condition at Faculty of Agriculture, Universiti Putra Malaysia from July 2014 to January 2015. The cultivated rice included the following: four rice ecotypes with a light brown pericarp colour called IR 402, IR409, IR420, IR425, five rice cultivars with a red pericarp colour called RP511, RP520, RP533, RP538 and RP544; seven rice cultivar with a black pericarp colour called RB211, RB218, RB222, RB225, RB233, RB246 and RB248. After harvest, the grains were dried to 13 ± 1% of moisture at a grain mass temperature below 40 °C. All paddy rice samples were dehulled and polished using rice dehusker and rice milling machine, set at 8% degree of milling, to obtain the milled rice bran. In order to separate the grains from the rice bran, they were sieved through 180 μm sieve (80 mesh). Rice bran was heated at 100 °C for 15 min in order to inactivate endogenous lipases.

### Extraction of free phenolics and flavonoids

Brown, red and black rice bran (0.5 g) were treated with 50 mL of acidified methanol solution (95% methanol: 1 M HCl 85:15, v/v). The mixture was homogenised using homogenizer for 5 min in an ice bath. Solutions were centrifuged at 2500*g* for 10 min and supernatants were removed. The filtered supernatants were concentrated by evaporation at 45 °C using hot plate. The concentrated filtrate was then diluted with 10 mL of acidified methanol and stored until analysis.

### Extraction of bound phenolics and flavonoids

The residue obtained from the free phenolics extraction was hydrolyzed with NaOH (40 mL, 2 M) at room temperature for 1 h with continuous shaking. Hexanes (10 mL) were used to extract lipids. The hydrolysate was then neutralised with 10 mL of 2 M HCL. Solution was transferred to separation funnel and was then extracted five times with ethyl acetate. The ethyl acetate layer (supernatants) were pooled and evaporated using hot plate (at 45 °C). Residue was dissolved in distilled water (10 mL) and then stored until analysis.

### Total phenolic content

Extracts (200 μL) were diluted in 20 mL of distilled water. Folin-Ciocalteu reagent (tenfold diluted; 1 mL) was added and the mixture was incubated in total darkness for 10 min at room temperature. After this time, sodium carbonate 7.5% (1 mL) was added and incubated for 30 min, then the absorbance of the solution was read at 765 nm using a spectrophotometer (UV2550, Shimadzu, Japan). Different concentrations of gallic acid were used to prepare a calibration curve. Results were expressed as milligram gallic acid equivalents (GAE)/100 g DM [18].

### Total flavonoid content

Extracts (1 mL) were mixed with NaNO_2_ solution (4 mL, 1:5, w/v) and incubated at room temperature for 6 min. 0.3 mL of AlCl_3_ solution (1:10, w/v) was added, the reagents were mixed well, and the reaction was allowed to stand for another 6 min. Immediately after that, 1M NaOH solution (2.0 mL) was added to each extract and incubated for 10 min at room temperature. The absorbance of the solutions was read at 510 nm using a spectrophotometer (UV2550, Shimadzu, Japan). Different concentrations of quercetin standard were used to prepare a calibration curve. Results were expressed as milligram quercetin equivalents (QE)/100 gDM [18].

### Estimation of total anthocyanin content (TAC)

Different rice bran samples (50 mg) were extracted with methanol/HCl (99:1 v/v) solute on at 4 °C for overnight. The observation of each sample were measured at 530 and 657 nm using a spectrophotometer. (UV-2120 Optizen, Mecasys, Korea), and relative anthocyanin levels were determined using the following formula:

1 $${\text{TAC}} = {\text{ optical density }}\left( {\text{OD}} \right) \, 530\,{\text{nm }} - \, \left( {0.25 \times {\text{ OD }}657\,{\text{m}}} \right) \, \times {\text{ extraction volume }}\left( {\text{mL}} \right) \, \times \, 1/{\text{weight of sample }}\left( {\text{g}} \right)$$

Cyanidin 3-glucoside was used as a standard and results were expressed as milligrams of cyanidin 3-glucoside equivalents (Cy3-GE)/100 gDM.

### Separation and analysis of flavonoids and phenolic acids

Ultra-high performance liquid chromatography (UHPLC, 1290 Infinity Quaternary LC System, Agilent, Santa Clara, CA, USA) was used to separate and identify the phenolics and flavonoids. The chromatographic system conditions were set as follows: mobile phase, 0.03 M orthophosphoric acid (A) and methanol HPLC grade (B); detector, UV 360 nm; column, C18 column (5.0 μm, 4.6 mm inner diameter [ID]  ×  250 mm); column oven temperature, 35 °C; and flow rate, 1.0 mL/min. Gradient elution was performed as follows: 0–10 min, 10% B; 10–15 min, 50% B; 15–20 min, 100% B; and finally 5 min for washing. Linear regression equations were calculated using Y  =  aX  ±  b, where X is the concentration of the related compound and Y the peak area of the compound obtained from UHPLC. The linearity was established by the coefficient of determination (R^2^) [9].

### Evaluation of antioxidant activity

#### Nitric oxide scavenging activity

Different rice bran extracts (3 mL) at different concentrations (50–250 μg/mL) was transferred to the test tubes. Thereafter, 2 mL of the reaction mixture [1.0 mM sodium nitroprusside (SNP) in 0.5 M phosphate buffer, pH 7.4] were added and mixed well. The mixture was incubated for 60 min at 37 °C. After incubation, Griess reagent (0.1% *α*-naphthyl-ethylenediamine in water and 1% H_2_SO_4_ in 5% H_3_PO_4_) was added to the mixtures. The absorbance of the samples was measured spectrophotometrically, at 540 nm (UV2550, Shimadzu, Kyoto, Japan). Gallic acid and ascorbic acid were used as a positive control [13]. Nitric oxide (NO) scavenging activity (%) was calculated by, using the formula:

2 $$\% {\text{ NO scavenging activity }} = \, \left[ {\left( {{\text{absorbance}}_{\text{control}} - {\text{ absorbance}}_{\text{sample}} } \right)/\left({\text{absorbance}}_{\text{control}} \right)} \right] \, \times \, 100$$

#### 1,1-Diphenyl-2-picrylhydrazyl (DPPH) assay

The DPPH assay was used in order to evaluate the free radical scavenging activity of free and bound extracts. DPPH was dissolved in methanol at a concentration of 100 μM. The DPPH solution (3 mL) was mixed with 3 mL of various concentrations (10, 20, 40, 80, and 160 μg/mL) of extracts and incubated in a dark room for 20 min at 27 °C. After incubation, the absorbance of the samples was read at 517 nm using a spectrophotometer (UV2550, Shimadzu, Japan) [13]. Gallic acid and ascorbic acid were used as positive controls. The scavenging activity was calculated using the following formula:

3 $$\% {\text{ inhibition }} = \left[ {{{\left( {{\text{absorbance}}_{{{\text{control}}}} - {\text{ absorbance}}_{{{\text{sample}}}} } \right)} \mathord{\left/ {\vphantom {{\left( {{\text{absorbance}}_{{{\text{control}}}} - {\text{ absorbance}}_{{{\text{sample}}}} } \right)} {\left( {{\text{absorbance}}_{{{\text{control}}}} } \right)}}} \right. \kern-\nulldelimiterspace} {\left( {{\text{absorbance}}_{{{\text{control}}}} } \right)}}} \right]\times100$$

### Evaluation of antiproliferative activity (MTT assay)

#### Cell culture and treatment

Human breast cancer cell lines MCF-7, MDA-MB-231, and MDA-MB-453 were purchased from the laboratory of Molecular Biomedicie, Institute Bio-sience, Universiti Putra Malaysia, Serdang, Selangor, Malaysia. Cells were cultured in RPMI 1640 media containing 10% fetal bovine serum (FBS). Cell lines were incubated overnight at 37 °C in 5% CO_2_ for cell attachment. The cells were maintained by sub-culturing in 25 cm^2^ tissue culture flasks. Cells growing in the exponential phase were used for cell viability assay.

#### TT (3-(4,5-dimethylthiazol-2-yl)-2,5-diphenyltetrazolium bromide) assay

The assay was conducted as follows: cancer cells were seeded in 96-well plates at a density of 1 × 10^4^ cells/well in 100 μL RPMI. After 24 h, the medium was removed and the cells were incubated for 3 days with RPMI in the presence or absence of various concentrations of brown, red and black rice bran extract (test extracts were prepared in 0.1% Dimethyl sulfoxide and serially diluted with media to obtain appropriate concentrations). Cells in the control group received only media containing 0.1% Dimethyl sulfoxide (DMSO). After incubation, the test compound containing media was removed and washed with 200 μL of PBS followed by addition of 20 μL of MTT reagent (5 mg/mL MTT in PBS) and incubated for 4 h at 37 °C. The medium was removed and 100 μL DMSO was added and the absorbance measured using a micro plate reader at 540 nm followed by the calculation of percentage viability 0.1% (v/v) DMSO in medium was used as negative control. Tamoxifen was used as positive control. The cell viability was determined using the formula:

4 $${\text{Viability }}\left( \% \right) = 100 - \left( {{\text{optical density of sample}}/{\text{optical density of control}}} \right) \times 100$$

5 $$\begin{gathered} {\text{Optical density of sample }} = {\text{ absorbance of cells treated with extract }} \\- {\text{ absorbance of cells treated with }}0.1\% {\text{ DMSO medium}} \end{gathered}$$

Optical density of control: absorbance of cells treated with 0.1% DMSO medium. Each point represents the mean of triplicate experiments [18].

### Statistical analysis

All data from the study were shown as mean ± SD of three replicates of each sample. Means were compared using analysis of variance, (ANOVA) using the Statistical Analysis System software (SAS 9.0, SAS.Institute, Cary, NC, USA). The data obtained were manipulated, to calculate statistical values such as means and standard deviations (SD) using Microsoft Excel (Microsoft Inc., Redmond, WA, USA). Group means were compared using Duncan’s tests. A value of p < 0.05 was considered to be statistically different.

## Results and discussion

### Phenolics and flavonoids content

The free, bound, and total phenolic acid contents in the bran fractions of sixteen different genotypes of pigmented rice are shown in Table 1. The free phenolic content in the bran fraction varied from 153.30 to 771.15 mg GAE/100 g DM. The bound phenolic content ranged from 102.05 to 443.55 mg GAE/100 g. The total phenolic content ranged from 269.85 to 1214.7 mg GAE/100 g DM. As shown in Table 1, black rice bran contained the highest contents of free, bound, and total phenolics (771.15, 443.55, and 1214.7 mg GAE/100 g DM, respectively), followed by red rice bran (579.46, 231.86, and 811.32 mg GAE/100 g DM, respectively) and light brown rice bran (329.65, 120.04, and 447.68 mg GAE/100 g DM, respectively).

**Table 1:** Free, bound and total content of phenolics, flavonoids and anthocyanins of sixteen different genotypes of rice with black, red and brown bran color

Rice bran colour	TPC (mg GAE/100 g DM)			TFC (mg QE/100 g DM)			TAC (mg Cy3-GE/100 g DM)		
	Free	Bound	Total	Free	Bound	Total	Free	Bound	Total
Black (RB211)	635.81^c^ ± 40.53	308.21^b^ ± 12.50	944.02^b^ ± 47.62	391.34^c^ ± 15.76	161.86^c^ ± 7.37	553.20^c^ ± 20.73	168.20^d^ ± 8.24	22.51^c^ ± 2.00	91 190.71^d^ ± 13.61
Black (RB218)	771.15^a^ ± 42.07	443.55^a^ ± 16.21	1214.70^a^ ± 48.71	526.68^a^ ± 21.65	297.20^a^ ± 10.82	823.88^a^ ± 38.59	256.11^a^ ± 10.44	38.51^a^ ± 3.66	294.62^a^ ± 12.05
Black (RB222)	613.04^c^ ± 36.25	265.44^c^ ± 10.67	878.48^c^ ± 41.47	348.57^d^ ± 14.17	119.09^d^ ± 4.29	467.66^e^ ± 22.74	150.20^e^ ± 6.79	20.37^c^ ± 2.54	170.57^e^ ± 8.64
Black (RB225)	703.16^b^ ± 37.71	295.56^b^ ± 11.47	998.72^b^ ± 46.32	378.69^c^ ± 12.62	149.21^c^ ± 6.35	527.90^d^ ± 24.61	188.37^c^ ± 7.22	26.18^b^ ± 3.06	214.55^c^ ± 9.65
Black (RB233)	684.86^b^ ± 30.15	297.26^b^ ± 11.88	982.12^b^ ± 44.62	480.39^b^ ± 18.26	250.91^b^ ± 10.82	731.30^b^ ± 33.60	201.63^b^ ± 10.19	29.91^b^ ± 3.17	231.54^b^ ± 11.39
Black (RB246)	585.25^d^ ± 26.37	257.65^c^ ± 15.21	842.90^d^ ± 39.52	340.78^d^ ± 15.05	111.30^d^ ± 4.25	452.08^e^ ± 19.73	69.20^g^ ± 4.55	14.84^d^ ± 1.61	84.04^f^ ± 4.75
Black (RB248)	616.87^c^ ± 31.25	289.27^b^ ± 13.27	906.14^c^ ± 43.77	372.40^c^ ± 12.69	142.92^c^ ± 7.28	515.32^d^ ± 20.26	144.30^f^ ± 6.30	18.70^c^ ± 1.22	163.00^e^ ± 7.26
Red (RP511)	579.46^d^ ± 25.17	231.86^c^ ± 11.54	811.32^e^ ± 40.39	314.99^d^ ± 14.42	85.51^e^ ± 2.05	400.50^g^ ± 16.30	63.21^g^ ± 3.65	14.66^d^ ± 1.59	77.87^g^ ± 5.28
Red (RP520)	462.71^e^ ± 22.61	135.11^d^ ± 7.32	597.82^g^ ± 22.40	218.24^e^ ± 10.65	238.76^b^ ± 10.22	457.00^e^ ± 19.62	46.63^h^ ± 391	5.25^f^ ± 0.72	51.88^h^ ± 3.92
Red (RP533)	475.70^e^ ± 19.38	148.10^d^ ± 6.48	623.80^f^ ± 29.68	231.23^e^ ± 9.72	101.75^d^ ± 2.88	332.98^h^ ± 15.27	48.91^h^ ± 3.88	5.61^f^ ± 0.62	54.52^h^ ± 4.06
Red (RP538)	482.79^e^ ± 20.16	155.19^d^ ± 6.89	637.98^f^ ± 30.04	238.32^e^ ± 10.16	110.32^d^ ± 3.74	348.64^h^ ± 14.66	51.20^h^ ± 2.76	10.62^e^ ± 1.52	61.82^h^ ± 3.33
Red (RP544)	569.39^d^ ± 22.54	241.79^c^ ± 10.42	811.18^e^ ± 38.52	324.92^d^ ± 15.32	95.44^e^ ± 2.40	420.36^f^ ± 16.62	60.16^g^ ± 4.21	12.27^d^ ± 1.38	72.43^g^ ± 5.27
Brown (IR402)	153.30^h^ ± 6.93	116.55^f^ ± 5.81	269.85^i^ ± 10.96	28.52^h^ ± 3.46	11.63^g^ ± 1.26	40.15 ^k^ ± 4.77	2.18^k^ ± 0.35	ND	2.18^k^ ± 0.41
Brown (IR409)	276.88^g^ ± 11.42	149.28^d^ ± 6.90	426.16^h^ ± 15.30	82.41^g^ ± 4.91	52.93^f^ ± 2.60	135.34^j^ ± 5.22	7.31^j^ ± 0.82	ND	7.31^j^ ± 0.64
Brown (IR420)	329.65^f^ ± 15.30	102.05^e^ ± 8.65	431.70^h^ ± 15.04	135.18^f^ ± 5.72	105.70^d^ ± 2.91	240.88^i^ ± 10.61	10.72^i^ ± 1.38	ND	10.72^i^ ± 1.12
Brown (IR425)	327.64^f^ ± 14.18	120.04^e^ ± 7.19	447.68^h^ ± 16.71	133.17^f^ ± 5.40	100.69^d^ ± 2.73	233.86^i^ ± 11.16	10.25^i^ ± 1.19	ND	10.25^i^ ± 0.98

Data are means of triplicate measurements ± standard deviation. Means not sharing a common single letter in each column for each measurement were significantly different at p < 0.05

*ND* not detected

The free flavonoid content in the bran fractions varied from 28.52 to 526.68 mg QE/100 g DM. The bound flavonoid content ranged from 11.63 to 297.20 mg QE/100 g, and the total flavonoid content ranged from 40.15 to 823.88 mg QE/100 g DM. As shown in Table 1, black rice bran possessed the highest free, bound, and total flavonoid contents (526.68, 297.20, and 823.88 mg QE/100 g DM, respectively), followed by red rice bran (324.92, 238.76, and 457.00 mg QE/100 g DM, respectively) and brown rice bran (135.18, 105.7, and 240.88 mg QE/100 g DM, respectively). In a recent study by Shen et al. [19] the free total flavonoid contents of white, red, and black rice were compared and it was found that the mean flavonoids content in white rice was lower than those in red and black rice.

The current results showed that the phenolic and flavonoid compounds in rice bran were mostly present in the free form, and this is an important issue for future studies. The bound forms of phenolics and flavonoids are covalently conjugated to the structures of the cell wall via ester bonds [20]. They cannot be directly digested and can survive gastrointestinal digestion to reach the colon intact. In the colon, they are broken down by the microflora and may release the bound phenolics to exert beneficial biological actions locally [17]. The current results are consistent with previous findings, in that phenolics and flavonoids in cereals were primarily distributed in the free form [21, 22]. Rice bran has attracted significant attention from consumers owing to its unique physiological functions and nutritional value. Some nutritional phytochemicals in rice bran primarily exist as glycosides linked to various sugar moieties or as other complexes linked to carbohydrates, lipids, organic acids, amines, and other phenols. Moreover, phytochemicals are commonly present in the bound form and as components of complex structures such as hydrolysed tannins and lignins [7].

### Anthocyanin content

Free and bound anthocyanin contents in sixteen different genotypes of pigmented rice are presented in Table 1. The total anthocyanins content in the free and bound form differed among the different genotypes of pigmented rice bran. The free and bound anthocyanin content in the bran fraction of sixteen different genotypes of pigmented rice ranged from 2.18 to 256.11 and 5.25 to 38.51 mg Cy_3_-GE/100 g DM, respectively. The highest anthocyanin concentration was detected in the free form. Black rice bran showed the highest content of total anthocyanins (294.62 mg Cy_3_-GE/100 g DM) followed by red (77.87 mg Cy_3_-GE/100 g DM) and brown rice (10.72 mg Cy_3_-GE/100 g DM). The bound form of anthocyanin was not detected in brown rice bran. It has been reported that the content of anthocyanin in rice is related to the expression levels of anthocyanin biosynthetic genes [23]. It was found that coloured rice exhibits stronger anthocyanin and antioxidant activities than those exhibited by non-coloured rice [24]. A recent study showed that the concentration of anthocyanin in black, blue, pink, purple, and red cereal grains was significantly dependent on the colour of the grain [25]. Moreover, the present findings showed that the anthocyanin content in rice correlated with the colour of the grain.

### Phenolics and flavonoids composition

Five phenolic compounds (protocatechuic acid, syringic acid, ferulic acid, cinnamic acid, and *p*-coumaric acid) and five flavonoid compounds (quercetin, apigenin, catechin, luteolin, and myrecitin) were detected in the free and bound fractions of three different pigmented rice bran (Table 2).

**Table 2:** Identified free, bound and total individual phenolics and flavonoids from black, red and brown rice bran

Rice bran	Black (RB218)			Red (RP511)			Brown (IR425)		
Phenolic acids and flavonoids	Free	Bound	Total	Free	Bound	Total	Free	Bound	Total
Protocatechuic acid	6.18^a^ ± 0.78	ND	6.18^a^ ± 0.63	5.31^b^ ± 0.74	ND	5.31^b^ ± 0.67	2.87^c^ ± 0.59	ND	2.87^c^ ± 0.39
Syringic acid	17.50^c^ ± 1.20	6.90^f^ ± 0.91	24.40^a^ ± 1.59	15.25^d^ ± 1.27	6.25^f^ ± 0.57	21.50^b^ ± 1.65	11.26^e^ ± 0.72	3.16^g^ ± 0.55	14.42^d^ ± 1.15
Ferulic acid	7.46^f^ ± 0.88	20.58^c^ ± 1.46	28.04^a^ ± 1.80	5.27 ^g^ ± 0.86	18.56^d^ ± 1.06	23.83^b^ ± 1.89	3.51 ^h^ ± 0.73	14.28^e^ ± 1.01	17.79^d^ ± 1.37
Cinnamic acid	19.98^b^ ± 1.38	5.55^e^ ± 0.69	25.53^a^ ± 1.92	15.33^c^ ± 1.19	ND	15.33^c^ ± 1.09	9.61^d^ ± 0.92	ND	9.61^d^ ± 0.84
*p*-coumaric acid	10.41^d^ ± 1.04	22.94^b^ ± 1.84	33.35^a^ ± 2.06	6.18^e^ ± 0.79	18.35^c^ ± 1.11	24.53^b^ ± 1.53	4.08^e^ ± 0.58	12.63^d^ ± 0.96	16.71^c^ ± 1.18
Quercetin	11.89^b^ ± 0.98	3.66^e^ ± 0.21	15.55^a^ ± 1.20	8.10^d^ ± 0.94	1.17^g^ ± 0.24	9.27^c^ ± 1.04	2.71^f^ ± 0.66	0.16^h^ ± 0.02	2.87^f^ ± 0.36
Apigenin	12.56^b^ ± 1.47	2.75^f^ ± 0.18	15.31^a^ ± 1.07	5.50^8^ ± 0.68	0.81^g^ ± 0.09	6.39^c^ ± 0.88	3.48^e^ ± 0.61	0.74^g^ ± 0.12	4.22^d^ ± 0.44
Catechin	15.64^b^ ± 1.16	6.41^f^ ± 0.80	22.05^a^ ± 1.32	12.66^c^ ± 0.92	3.24^g^ ± 0.46	15.90^b^ ± 1.02	7.27^e^ ± 0.79	1.69^h^ ± 0.25	8.96^d^ ± 0.89
Luteolin	10.72^a^ ± 0.81	ND	10.72^a^ ± 0.91	7.74^b^ ± 0.81	ND	7.74^b^ ± 0.82	2.35^c^ ± 0.24	ND	2.35^c^ ± 0.30
Myrecitin	12.85^a^ ± 1.15	ND	12.85^a^ ± 0.88	12.82^a^ ± 0.90	ND	12.82^a^ ± 0.94	5.68^b^ ± 0.38	ND	5.68^b^ ± 0.72

Data are means of triplicate measurements ± standard deviation. Means not sharing a common single letter in each row for each measurement were significantly different at p < 0.05

*ND* not detected

Protocatechuic acid only existed in free fractions, with contents ranging from 2.87 to 6.18 mg/100 g DM and the highest content (p < 0.05) was found in black rice bran. Syringic acid existed in both the free and bound fractions, with contents ranging from 11.26 to 17.5 and from 3.16 to 6.9 mg/100 g DM, respectively. Black rice bran contained the highest content of free and bound syringic acid. Ferulic acid existed in both the free and bound fractions, with contents ranging from 3.51 to 7.56 and from 14.28 to 20.58 mg/100 g DM, respectively. As shown in the data, the highest concentration of ferulic acid was detected in the bound form Black rice bran showed the highest content of free and bound ferulic acid. The contents of the free form of cinnamic acid were between 9.61 and 19.98 mg/100 g DM, and the bound form was only detected in black rice bran (5.55 mg/100 g DM). *P*-coumaric acid existed in both the free and bound forms, with content ranging from 4.08 to 10.41 and from 12.63 to 22.94 mg/100 g DM, respectively. Similar to that observed with ferulic acid, the highest concentration of p-coumaric acid was observed in the bound form. Black rice bran contained the highest content of free and bound p-coumaric acid. These data suggest that the contents of *p*-coumaric acid and ferulic acid were relatively high compared with the contents of other phenolic compounds in brown, red, and black rice bran. The highest concentration of p-coumaric acid and ferulic acid was found in the bound form.

Among the five flavonoid compounds identified in the three different pigmented rice bran, quercetin, apigenin, and catechin existed in the free and bound form but luteolin and myrecitin were only detected in the free form. The contents of quercetin in the free and bound form ranged from 2.71 to 11.89 mg/100 g DM and from 0.16 to 3.66 mg/100 g DM, respectively. Apigenin existed in both the free and bound forms, with contents between 3.48 and 12.56 and 0.74 and 2.75 mg/100 g DM, respectively. Catechin also existed in both the free and bound forms, with contents between 7.27 and 15.64 and 1.69 and 6.41 mg/100 g DM, respectively. Luteolin and myrecitin only existed in the free fractions, with contents ranging from 2.35 to 10.72 mg/100 g DM and 5.68 to 12.85 mg/100 g DM, respectively.

Black rice bran contained the highest content of all the identified flavonoids in both the free and bound forms, followed by red and brown rice bran. Ferulic acid and *p*-coumaric acid were the most abundant phenolic compounds in brown, red, and black rice bran extracts. Further, catechin and myrecitin were the most abundant flavonoid compounds in brown and red rice bran, while apigenin and quercetin were the most abundant flavonoid compounds in black rice bran Zhou et al. [26] showed that brown rice contained high levels of ferulic and p-coumaric acid and low levels of gallic, vanillic, caffeic, and syringic acids, which is consistent with the findings of the present study. Arabinoxylans are present in the walls of aleurone cells, indicating that they contain high levels of ferulic acid In addition, the benefits of bound ferulic and *p*-coumaric acids, which are mainly present in rice bran, may be site-specific i.e. more effective in the colon. Bound forms of flavonoids and phenolic acids are covalently conjugated to the structures of the cell wall via ester bonds. The phytochemical constituents and their quality in rice grain vary considerably and this may be attributed to several factors, such as agronomic activities, environmental conditions, and genetic factors [27]. The milling fractions obtained from different rice varieties will exhibit different chemical composition and nutritional values. The chemical composition in rice bran, polished rice, and whole brown rice grain are also different within one variety.

### Antioxidant activities

#### Nitric oxide (NO) scavenging activity

**Fig. 1:**
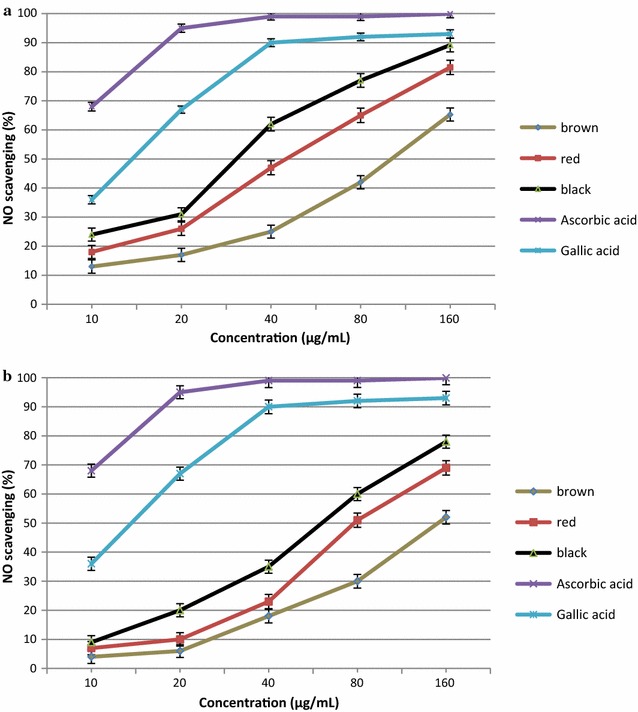
Nitric oxide radical scavenging activity of free (**a**) and bound (**b**) fraction of black, red and brown rice bran extracts

Nitric oxide scavenging activity of brown, red, and black rice bran fraction at different concentrations is shown in Fig. 1. As shown in Fig. 1, as rice bran concentration increased from 10 to 160 μg/mL, NO scavenging activity of the free and bound fractions increased significantly (p < 0.05). The NO scavenging activity of the rice bran of three different genotypes ranged from 4.0 to 89.2%. The NO scavenging activity in the free and bound fractions ranged from 13.4 to 89.2 and 4.0 to 78.0%, respectively. Significant difference (p < 0.05) in NO scavenging activity was found among the different coloured genotypes of rice. Black rice bran demonstrated the highest NO-scavenging activity followed by red and brown rice bran extracts. The NO-scavenging activity of the free fraction was higher than that of the bound fractions at all concentrations (10–160 μg/mL).

The antioxidant activities of all rice bran were lower than those of ascorbic acid and gallic acid. The IC_50_ values for NO scavenging activity of the free fractions of black, red, and brown rice bran were 32.0, 44.5, and 112 μg/mL, respectively. While, the IC_50_ values of the bound fractions of black, red, and brown rice bran were 65.7, 78.2, and 150.4 μg/mL, respectively. IC_50_ values of ascorbic acid and gallic acid were < 10 and 14.8 μg/mL, respectively. A lower IC_50_ value represents a stronger free radical inhibitor (strong free radical inhibitors are active at low concentrations). The free fractions of rice bran extracts exhibitted strong NO radical scavenging activity with low IC_50_ values, indicating that the antioxidant activity of free compounds in black, red, and brown rice bran was higher than that of bound compounds in these rice genotypes. It has been reported that grains with red and black pericarp demonstrated higher antioxidant activity than those demonstrated by grains with light brown pericarp [28]. Rice bran, though in small amounts, is rich in antioxidants; hence, removal of the bran during the production of polished rice leads to lower antioxidant activity. This indicates that black rice is a good source of antioxidants when compared with brown rice, which is generally consumed in our diet. Nowadays, more rice varieties have been developed as healthy foods and have gained increasing popularity with consumers [21]. Since nutritional imbalance in the diet can cause diseases such as obesity, diabetes, cardiovascular disease, and cancer, action is needed to promote whole rice as a “nutritious health food” and as a normal part of everyday meal consumption [8]. In addition, safety concerns over the use of synthetic antioxidants have led to increasing interest from the food industry in identifying naturally occurring antioxidants in basic raw food materials. Rice bran, with its low cost, has great potential for applications in the food and pharmaceutical industries as a rich source of natural antioxidants [22].

#### DPPH activity

Various mechanisms, such as free radical-scavenging, reducing capacity, metal ion-chelation, and inhibition of lipid peroxidation, have been studied to explain how rice bran extracts could be used as effective antioxidants [13, 29]. DPPH radical-scavenging assays are based on the transfer of electrons from a donor molecule to the corresponding radical. This method is the simplest method to measure the ability of antioxidants to intercept free radicals.

**Fig. 2:**
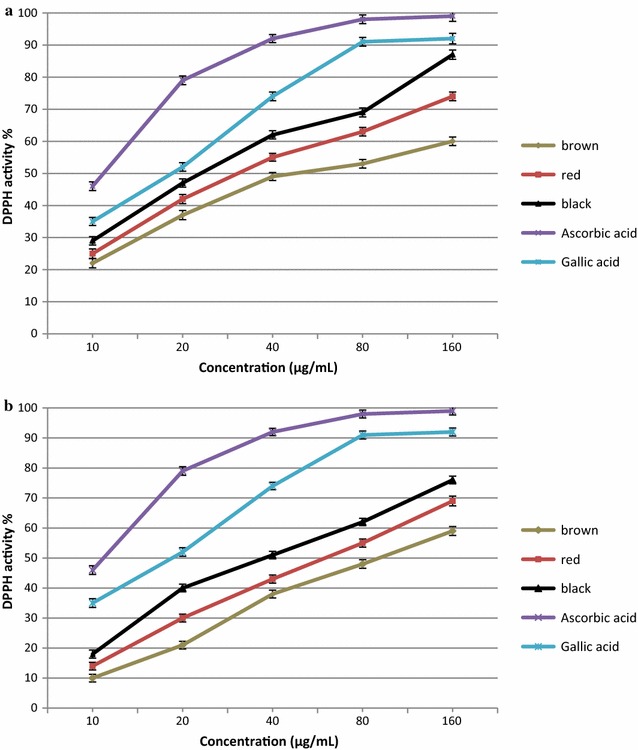
DPPH radical scavenging activity of free (**a**) and bound (**b**) fraction of black, red and brown rice bran extracts

The DPPH radical-scavenging effects of all rice bran extracts (free and bound) increased with increasing concentration (Fig. 2). DPPH activity was significantly influenced (p < 0.05) by the colour of the rice bran. Black rice bran extract demonstrated the highest DPPH activity followed by red and brown rice extracts. The DPPH activity of the rice bran of three different pigmented rice bran ranged from 10.7 to 87.9%. The DPPH activity in the free and bound fractions ranged from 22.6 to 87.9 and 10.7 to 76.1%, respectively. Black rice bran exhibited the highest DPPH activity, followed by red and brown rice bran extracts. DPPH activity in free fractions was higher than that of bound fractions at all concentrations (10–160 μg/mL). The DPPH activity of all rice bran extracts was lower than those of the positive controls (ascorbic acid and gallic acid). The IC_50_ values of the free fractions of black, red, and brown rice bran against DPPH activity were 25, 32, and 51 μg/mL, respectively. IC_50_ values for DPPH radical scavenging activity of the bound fractions in black, red, and brown rice bran extract were 39.1, 64.7, and 87.1 μg/mL, respectively. The lowest IC_50_ value was obtained in the free form, indicating that free compounds exhibit potent antioxidant property compared to that of bound compounds. The IC_50_ values of ascorbic acid and gallic acid were 12.4 and 19.2 μg/mL, respectively.

The highest DPPH activity and the lowest IC_50_ value of the rice bran extracts was observed with black rice, which contained the highest content of phenolic and flavonoid compounds. The higher levels of secondary metabolites (flavonoids, phenolic acids, and anthocyanins) in black rice compared to those of red and brown rice might be responsible for the high antioxidant activity. Previous studies have reported that the concentration of total phenolics and flavonoids in rice grains positively correlated with the antioxidant activity [18, 30, 31]. Oki et al. [32] reported that in red pericarp grains, a strong correlation between antioxidant activity and the content of proanthocyanidins was observed; however, in the case of black pericarp grains, the correlation was dependent on the content of anthocyanins. These results suggest that phenolic compounds were primarily responsible for the antioxidant activity of rice grains [33].

### Antiproliferative activity

**Fig. 3:**
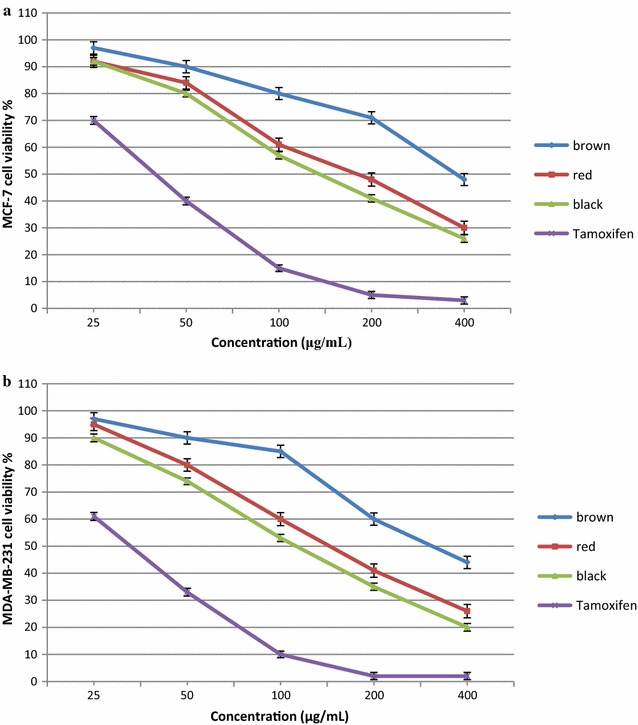
Antiprolifrative activity of black, red and brown rice bran extracts against MCF-7 (**a**) and MDA-MB-231 (**b**) cell lines. Bars represent standard error of the means

The free fractions of black, red, and brown rice bran extracts were subjected to antiproliferative assays since it demonstrated the highest antioxidant activity and phytochemical content. The antiproliferative activity of the bran extracts of black, red, and brown rice (25–400 μg/mL) was evaluated against the breast cancer cell lines MCF-7 and MDA-MB-231 (Fig. 3). The MTT assay indicated that black, red, and brown rice bran reduced the viability of MCF-7 and MDA-MB-231 cells in a dose-dependent manner. The IC_50_ values differed significantly (p  <  0.05) among the different pigmented rice bran. The black rice bran extracts exhibited potent antiproliferative activity, with IC_50_ values of 148.6 and 119.2 mg/mL against MCF-7 and MDA-MB-231 cells, compared to those of red rice bran extracts (175 and 151 mg/mL, respectively) and brown rice bran (382.3 and 346.1 mg/mL, respectively) (Fig. 3). The biological properties and reactions to certain agents differ between different breast cancer cell lines. MDA-MB-231 cells were the most sensitive to treatment with different pigmented rice bran followed by the MCF-7 cell line; therefore, their pro-apoptotic responses to different pigmented rice bran were analysed. The IC_50_ values of all the extracts were higher that of than tamoxifen (MDA-MB-231 = 40.2 mg/mL; MCF-7 = 22.8 mg/mL), which is a breast cancer drug. Previous studies have reported the anticancer activity of brown rice bran on breast cancer cell lines such as MDA-MB-468 [34, 35], MCF-7 [36] and MDA-MB-231 [37]. It has been reported that the cytotoxic activity of rice bran against breast cancer cell lines is influenced by the variety of rice, growing conditions, cultivation process, and the type of cancer cell lines [35, 37].

**Fig. 4:**
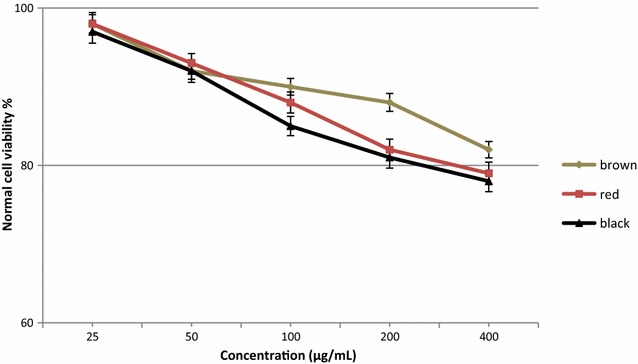
Toxicity effect of black, red and brown rice bran extracts against normal cell line. Bars represent standard error of the means

Extracts of black, red, and brown rice bran at concentrations between 25 and 400 μg/mL did not exert toxic effect against normal cells (MCF-10A), with viability between 77 and 98% (Fig. 4). In food supplements, one ingredient may provide the desired therapeutic benefits while others may exert toxic effects. The American National Cancer Institute recommends that crude herbal extracts that do not reduce the viability of normal cells below 76% is safe for human consumption [8]. In the current study, black rice bran contained the highest content of secondary metabolites, including anthocyanins, phenolics, and flavonoids, and exhibited the highest antioxidant and antiproliferative activity. Therefore, in general, it appears that the antioxidant and antiproliferative activities of black rice bran are attributed to the high level of phytochemicals. However, further research is needed to understand the relationship between these phytochemicals and the antiproliferative activity in black rice bran extracts. The current results corroborate the findings of a great deal of previous work in this field.

## Conclusions

In this study, the phenolic and flavonoid compounds and their free and bound fractions as well as the antioxidant and antiproliferative activities of black, red, and brown rice bran were reported. The antioxidant activity and the content of free and bound phenolics, flavonoids, and anthocyanins, significantly differed among the different pigmented rice bran. In free fractions, significant amount of flavonoids and phenolics were determined. Cinnamic acid and *p*-coumaric acid were identified in the bound fractions and the most abundant phenolics. Catechin and myrecitin were abundant in red and brown rice bran, while quercetin and catechin were abundant in black rice bran compared to the levels of other flavonoids. Black rice bran contained the highest phytochemical constituents and exhibited strong antioxidant and antiproliferative activity against breast cancer cells, followed by red and brown rice. It can be concluded that black rice bran appears to be a rich source of antioxidants and has potential application in the food industry. These findings provide important information to improve human health by encouraging the consumption of black rice bran and its use in food product development.
